# Thermostable Carbonic Anhydrases in Biotechnological Applications

**DOI:** 10.3390/ijms160715456

**Published:** 2015-07-08

**Authors:** Anna Di Fiore, Vincenzo Alterio, Simona M. Monti, Giuseppina De Simone, Katia D’Ambrosio

**Affiliations:** Istituto di Biostrutture e Bioimmagini-CNR, via Mezzocannone 16, 80134 Napoli, Italy; E-Mails: vincenzo.alterio@cnr.it (V.A.); marmonti@unina.it (S.M.M.); gdesimon@unina.it (G.D.S.)

**Keywords:** CO_2_ capture process, thermostable enzyme, carbonic anhydrases, protein engineering

## Abstract

Carbonic anhydrases are ubiquitous metallo-enzymes which catalyze the reversible hydration of carbon dioxide in bicarbonate ions and protons. Recent years have seen an increasing interest in the utilization of these enzymes in CO_2_ capture and storage processes. However, since this use is greatly limited by the harsh conditions required in these processes, the employment of thermostable enzymes, both those isolated by thermophilic organisms and those obtained by protein engineering techniques, represents an interesting possibility. In this review we will provide an extensive description of the thermostable carbonic anhydrases so far reported and the main processes in which these enzymes have found an application.

## 1. Introduction

Increased atmospheric carbon dioxide levels have been correlated with global warming. CO_2_ can be emitted from various sources, but mostly from the burning of fossil fuels [1,2,3]. To reduce the levels of atmospheric CO_2_, the use of renewable source of energy and the shift toward activities requiring less energy have been encouraged all around the world. However, considering the present energy demand, fossil fuels are difficult to completely substitute in the near future. Therefore, in the transition period, it is necessary to develop methods to reduce the concentration of CO_2_ in the atmosphere. One practical and effective approach is to capture CO_2_ from flue gas. Once that CO_2_ is sequestered, it can be either pressurized to a liquid or chemically converted to a stable compound, which can be subsequently stored in the ocean or underground [4,5]. It is also possible to convert the captured CO_2_ into various beneficial by-products which include acrylates, polycarbonates, stable carbonate storage polymers, methane and building materials [6,7,8].

Different techniques have been developed to perform CO_2_ capture [9]; however, the majority of these methods are too expensive and of limited efficiency. Thus, some biological methodologies, also called “bio-mimetic” CO_2_ capture systems, have been implemented as more economic and more sustainable technologies. They are based on the use of enzymes involved in CO_2_ biological processes, occurring naturally in living organisms. Carbonic anhydrases (CAs) (EC 4.2.1.1), which catalyze the reversible hydration of the CO_2_ molecule (CO_2_ + H_2_O ↔ HCO_3_^−^ + H^+^), can be efficiently used in these processes [10].

CAs are ubiquitous metallo-enzymes present in prokaryotes and eukaryotes, being encoded by six distinct, evolutionarily unrelated gene families: the α-CAs (present in vertebrates, eubacteria, algae and cytoplasm of green plants), β-CAs (predominantly in eubacteria, algae and chloroplasts of both mono- as well as dicotyledons), γ-CAs (mainly in Archaea and some eubacteria), δ- and ζ-CAs (both discovered in marine diatoms), and the recently identified η-CAs (present in different *Plasmodium* spp*.*) [10,11,12]. To perform catalytic activity, CAs need the presence in their active site of a metal ion (generally Zn^2+^) coordinated by three residues, which can be hystidines, cysteines or glutamine, depending on the enzyme class. The catalytic mechanism for the CO_2_ hydration reaction, studied in detail for the α-, β- and γ-class, consists of two steps [13,14]. In the first step a zinc-bound hydroxide leads the nucleophilic attack on a CO_2_ molecule with formation of bicarbonate bound to the zinc ion, which is then substituted by a water molecule (Equation (1)). The second step, the rate limiting one, consists of the regeneration of the enzyme reactive species, the zinc-bound hydroxide, via a proton transfer reaction, which occurs from the zinc-bound water molecule to the external buffer (Equation (2)). This process is generally assisted by an enzyme residue which acts as proton shuttle; in most human isoforms this residue is a histidine [15,16].

(1) $${\text{CO}}_{2}+{\text{EZnOH}}^{-}↔{{\text{EZnHCO}}_{3}}^{-}\overset{{\text{H}}_{2}\text{O}}{↔}{\text{EZnH}}_{2}\text{O}+{{\text{HCO}}_{3}}^{-}$$

EZnH_2_O + B ↔ EZnOH^−^ + BH^+^ (2)

The last years have seen an increasing interest in using CAs in CO_2_ capture and storage processes [17,18,19]; however, this use is greatly limited by the harsh conditions required in these processes, *i.e.*, high temperatures and high concentrations of organic ions and metals [20,21,22]. In this context the employment of thermostable enzymes may represent an interesting possibility. These enzymes can be obtained through either isolation from microorganisms living at high temperature, *i.e.*, thermophiles, or protein engineering techniques applied to mesophilic proteins.

In this review an extensive overview of the thermostable CAs so far reported and their biotechnological applications will be provided. In particular, the first part of the paper will be dedicated to the description of the processes in which the involvement of these enzymes has been described, highlighting the most recent advances in such field. The second part will be instead focused on the biochemical and structural data currently available in literature on thermostable CAs, both those isolated from thermophilic organisms and those obtained by protein engineering techniques.

## 2. Thermostable CAs in CO_2_ Capture and Storage Processes

**Figure 1:**
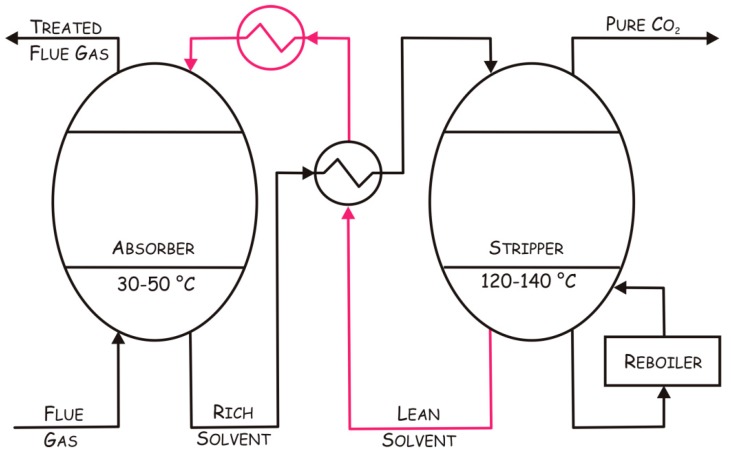
Schematic representation of a chemical absorption process. Red lines indicate the absorber regeneration path.

The term “carbon capture and storage (CCS)” is generally used to describe the set of technologies aimed at capturing CO_2_ produced by major emitters and its long term storage. Different techniques have been developed in order to capture CO_2_, such as absorption into a liquid, adsorption on a solid, gas phase separation, and membrane systems [9], and among these the use of thermostable CAs has been described for chemical absorption [23]. This process involves the reaction of CO_2_ with a chemical solvent to form an intermediate compound, which is then treated with heat, regenerating the original solvent and a CO_2_ stream. In particular, in a typical chemical absorption process (Figure 1), the flue gas containing CO_2_ enters from the bottom into a packed absorber column at low temperature (30–50 °C) and then contacts a CO_2_ absorber; after absorption, the CO_2_-rich solvent is sent to a stripper operating at high temperatures (120–140 °C) to recover the absorber and CO_2_. After regeneration, the CO_2_-lean solvent is pumped back to the absorber for cyclic use, while the pure CO_2_ is compressed for the subsequent transportation and storage [23,24]. The main energy cost associated to this process is due to the heat required for the desorption and the pumping of the absorbing solution around the system [25].

Alkanolamines are among the most widely used absorbers for CO_2_ capture [26]; they include primary, secondary and tertiary amines, such as monoethanolamine (MEA), diethanolamine (DEA) and *N*-methyldiethanolamine (MDEA) [27].

The proposed mechanism of CO_2_ absorption by alkanolamines is described by the Equations (3)–(6):

R_1_R_2_NH + CO_2_ ↔ R_1_R_2_NH^+^COO^−^ (3)

R_1_R_2_NH^+^COO^−^ + R_1_R_2_NH ↔ R_1_R_2_NCOO^−^ + R_1_R_2_NH_2_^+^ (4)

R_1_R_2_NCOO^−^ + H_2_O ↔ R_1_R_2_NH + HCO_3_^−^ (5)

R_1_R_2_R_3_N + H_2_O + CO_2_ ↔ R_1_R_2_R_3_NH^+^ + HCO_3_^−^ (6)

where R_1_ is always an alkyl group, R_2_ is a hydrogen atom for primary amines and an alkyl group for secondary and tertiary amines and R_3_ is an alkyl group. In the case of primary and secondary amines the reaction with CO_2_ proceeds via a zwitterion mechanism. Initially, one amine group interacts with CO_2_ producing a zwitterion intermediate (Equation (3)), which reacts with a base, *i.e.*, a second amine group, generating a carbamate (Equation (4)). Finally, the interaction of the latter compound with a water molecule leads to bicarbonate (Equation (5)). On the other side, the mechanism involving tertiary amines is essentially a base-catalyzed hydration of CO_2_ and implies the formation of bicarbonate directly, without passing through the carbamate (Equation (6)) [28,29,30,31]. Several studies demonstrated that the use of tertiary amines in chemical absorption processes is more advantageous with respect to the use of primary and secondary amines, since they have a lower heat of regeneration [32] and a higher capture capacity. Indeed, in tertiary amines 1 mole of amine is required to absorb 1 mole of CO_2_, while primary and secondary amines load 0.5 mole of CO_2_ per mole of amine [33]. However, the reaction of CO_2_ with tertiary amines is significantly slower than that with primary and secondary amines [34]. Thus, the use of CAs has been proposed to increase the CO_2_ absorption rate [35], facilitating the intermolecular transfer of protons [36].

Considering the high temperature used in this process, several heat-stable CAs have been employed, such as the CAs isolated from *Sulfurihydrogenibium yellowstonense* YO3AOP1 (SspCA) [37,38] and from *Caminibacter mediatlanticus* (CmCA), both belonging to the α class [39,40]. Moreover, two highly stable variants of β-CAs from *Desulfovibrio vulgaris* (DvCA) [41] and *Methanobacterium thermoautotrophicum* (Cab) [42], obtained by protein engineering techniques, have also been tested for their capability to accelerate CO_2_ absorption in alkaline solvents.

Transportation of the pure CO_2_ for subsequent storage also requires expensive technology. In this context the CA-catalyzed CO_2_ capture and its direct storage as CaCO_3_, which can be safely returned to the environment, is a very promising approach [20]. This method involves a number of steps, which are summarized below:

CO_2(g)_ ↔ CO_2(aq)_ (7)

CO_2(aq)_ + H_2_O ↔ H_2_CO_3_ (8)

H_2_CO_3_ ↔ H^+^ + HCO_3_^−^ (9)

HCO_3_^−^ ↔ H^+^ + CO_3_^2−^ (10)

CO_3_^2−^ + Ca^2+^ ↔ CaCO_3_↓ (11)

**Figure 2:**
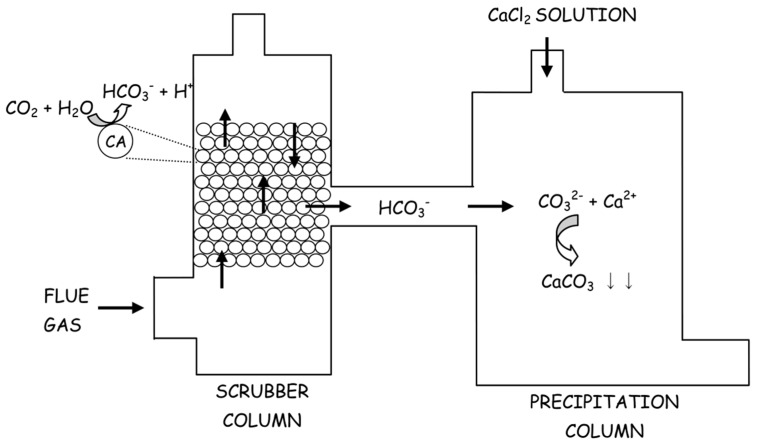
Schematic representation of CA-based CO_2_ capture system and its storage as CaCO_3_. CO_2_ was sequestered in the scrubber column and the obtained carbonate ions were transformed in CaCO_3_ in presence of CaCl_2_ in the precipitation column.

The first step involves the dissolution of gaseous CO_2_ (Equation (7)) and its consequent reaction with water forming carbonic acid (Equation (8)), which then ionizes in bicarbonate (Equation (9)) and carbonate ions (Equation (10)). Finally, in the presence of Ca^2+^ ions calcium carbonate is formed and precipitates (Equation (11)). Since hydration of CO_2_ (Equation (8)) constitutes the rate-limiting step of this process, starting from 2001 CAs have been used to accelerate this reaction [20]. The overall process of is schematically depicted in Figure 2.

Even if several studies reported the use in this process of CAs isolated from mesophilic microorganisms, such as *Serratia* sp*.* ISTD04 [43] and *Citrobacter freundii* [44], only recently two CAs isolated from thermophilic bacteria, *Persephonella marina* and *Thermovibrio ammonificans*, have been utilized [45,46,47,48,49].

In both CCS technologies above described, the utilization of free CAs in solution has some disadvantages, such as a low enzyme stability, a limited repeatable usage, and the impossibility of enzyme recovery from the reaction environment.

These disadvantages can be eliminated by immobilizing the enzyme within solid supports. Immobilization can be achieved both by physical or chemical methods. In the first case it involves the absorption of the enzyme onto a water insoluble matrix or supporting material, while in the second case it consists of the formation of covalent bonds between a water insoluble matrix and the enzyme [50]. So far a number of matrices have been used, such as metal-based nanoparticles [51], chitosan beads [52] and polyurethane [53], but only few thermostable CAs have been immobilized. Among these, SspCA has been immobilized on a polyurethane foam (HYPOL2060) [54] and CmCA on a hollow fibers membrane [40], generating in both cases highly stable and active enzymes.

Following immobilization unwanted release of enzyme from reactor surface can happen; thus, to develop more efficient immobilization procedures, a recent patent reports the utilization of two thermostable γ-CAs isolated from *Methanosarcina thermofila* and *Pyrococcus horikoshii*, respectively [55]*.* In particular, this study describes the formation of γ-CA nanoassemblies, where individual enzymes are connected each other and make multiple linked interactions with the reactor surface. This can be achieved by mutating specific enzyme residues to cysteines, in order to introduce sites for biotinylation, thus allowing the subsequent formation of stable nanostructures by cross-linking of biotinylated-γ-CAs with streptavidin tetramers [55]. Further addition of an immobilization sequence at amino- or carboxy-terminus also allows for a controlled and reversible immobilization of the γ-CA to a functionalized surface.

## 3. Carbonic Anhydrases Isolated from Thermophilic Bacteria

### 3.1. α-Carbonic Anhydrases

Although in the past only a few studies have reported on thermostable α-CAs, and most of them has been described in patents [40,47,56], recently numerous data on these enzymes have been published in open scientific literature. In particular, four highly thermostable α-CAs, isolated from *Sulfurihydrogenibium yellowstonense* YO3AOP1 (SspCA) [57,58], *Sulphurihydrogenibium azorense* (SazCA) [59,60,61], *Thermovibrio ammonificans* (TaCA) [62], and *Persephonella marina* EX-H1 (PmCA) [46], have been extensively studied both biochemically and kinetically.

SspCA, the first of these enzymes to be kinetically characterized, showed a very high catalytic activity for the CO_2_ hydration reaction (*k*_cat_ = 9.35 × 10^5^ s^−1^; *k*_cat_/*K*_M_ = 1.1 × 10^8^ M^−1^·s^−1^ at 20 °C and pH 7.5), comparable to that of human carbonic anhydrase II (hCA II), the most active human CA isoform (see Table 1) [57,58]. This enzyme also presented esterase activity, and a thermoactivity analysis revealed an optimum working temperature of 95 °C [57].

**Table 1:** Kinetic parameters for the CO_2_ hydration reaction catalyzed by thermostable CAs. Data on hCA II have been added for comparison.

Enzyme	Class	*k*_cat_ (s^−1^)	*k*_cat_/*K*_M_ (M^−1^·s^−1^)	Ref.
hCA II	α	1.40 × 10^6^	1.5 × 10^8^	[63]
SspCA	α	9.35 × 10^5^	1.1 × 10^8^	[57]
SazCA	α	4.40 × 10^6^	3.5 × 10^8^	[61]
TaCA	α	1.60 × 10^6^	1.6 × 10^8^	[62]
PmCA	α	3.2 × 10^5^	3.0 × 10^7^	[46]
Cab	β	1.7 × 10^4^	5.9 × 10^6^	[64]
MtCam (expressed in *E. coli* and purified aerobically)	γ	6.8 × 10^4^	3.1 × 10^6^	[65]
MtCam (expressed in *E. coli* and purified anaerobically)	γ	24.3 × 10^4^	5.4 × 10^6^	[65]
MtCam (expressed in *M. acetivorans* and purified anaerobically)	γ	23.1 × 10^4^	3.9 × 10^6^	[66]

Thermal stability studies highlighted for SspCA an extraordinary resistance to high temperatures; indeed, it is able to retain CO_2_ hydration activity after incubation at 100 °C for 3 h, while its human homologues, hCA I and hCA II, are inactivated at temperature higher than 60 °C for each incubation time tested [67]. Moreover, long-term stability studies showed that SspCA has a half-life of 53 and 8 days at 40 and 70 °C, respectively (Table 2), and that after incubation at these temperatures for 28 days the enzyme still presents a significant residual activity (74% at 40 °C and 10% at 70 °C) [68]. Finally, the effect of the immobilization within a polyurethane (PU) foam on SspCA kinetic properties and its long-term stability has also been evaluated, showing that after immobilization the enzyme is still active and stable up to 50 h at 100 °C [54].

**Table 2:** Long-term stability of some thermostable α-CAs at different temperatures. Data on bovine CA II (bCA II) have also been reported for comparison [45].

Temperature (°C)	Half-Life (Days)			
	bCA II	SspCA	TaCA	PmCA
40	6	53	152	75
60	n.d.	n.d.	77	29
70	<1	8	n.d.	n.d.

**Figure 3:**
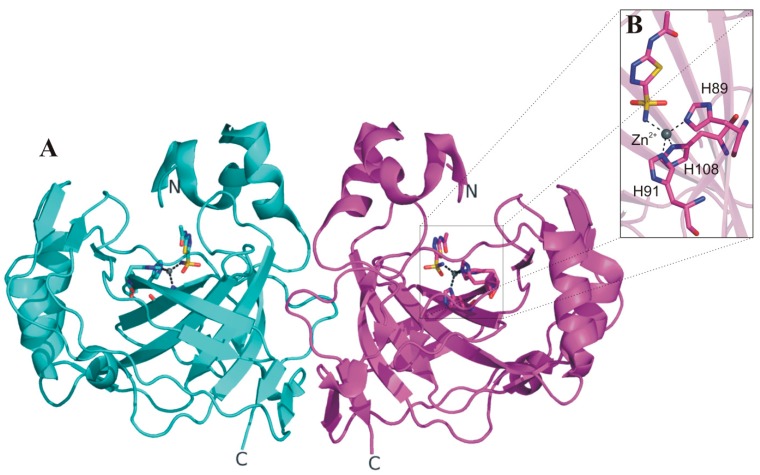
(**A**) SspCA dimer structure (PDB code 4G7A) with one monomer shown in cyan and the other one in magenta; (**B**) enlarged view of SspCA active site showing the zinc ion coordination.

The availability of the SspCA crystallographic structure provided critical information to clarify the molecular basis of its exceptional thermostability [67]. In particular, these studies showed that SspCA, similarly to the previously studied α-CAs [10], presents a fold characterized by a central ten-stranded β-sheet surrounded by several helices and additional β-strands (Figure 3A). The active site is found in a deep conical cavity which extends from the protein surface to the center of the molecule, with the catalytic zinc ion positioned at the bottom of this cavity. The metal ion is tetrahedrally coordinated by three histidine residues (His89, His91 and His108) and by the nitrogen atom of the inhibitor acetazolamide (AZM), which was co-crystallized with the enzyme (Figure 3B). Interestingly, SspCA forms a dimer characterized by a large interface area (Figure 3A), and stabilized by several polar and hydrophobic interactions. This dimeric arrangement is a peculiar feature of all bacterial α-CAs so far structurally characterized [62,69,70].

A detailed structural comparison of SspCA with its mesophilic homologues hCA I and hCA II suggested that a higher content of secondary structural elements (enhanced compactness), an increased number of charged residues on the protein surface and a greater number of ionic networks could be the key features responsible of its elevated thermostability [67].

SazCA, the other thermostable α-CA isolated from a *Sulphurihydrogenibium* species, has been demonstrated to be the most catalytically active CA ever investigated so far (Table 1) [61]. Indeed, its CO_2_ hydration activity, measured by a stopped-flow assay method at 20 °C and pH 7.5, is 2.33 times higher than that of the highly active hCA II, with a *k*_cat_ value of 4.40 × 10^6^ s^−1^ and a *k*_cat_/*K*_M_ value of 3.5 × 10^8^ M^−1^·s^−1^ [61]. Thermoactivity studies revealed that this enzyme presents esterase activity in a temperature range of 0 to 100 °C, with an optimum working temperature at 80 °C. Moreover, thermal stability studies showed that SazCA is able to retain CO_2_ hydration activity after incubation at 100 °C for 3 h, even though it is less stable than SspCA [61].

**Figure 4:**
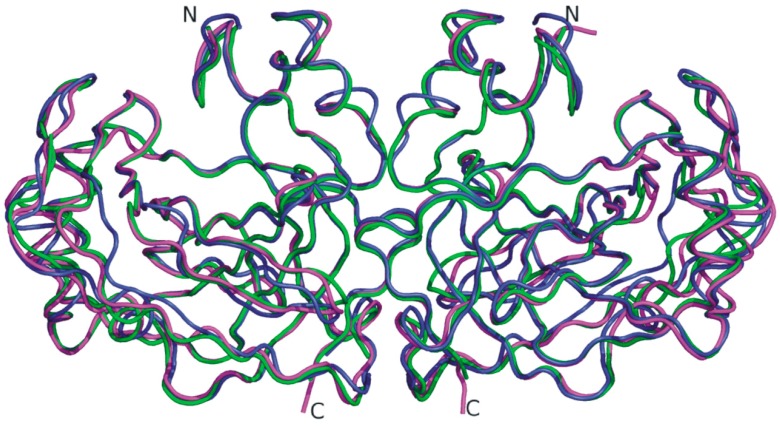
Superposition of the dimeric structure of SazCA (PDB code 4X5S, green) with those of SspCA (PDB code 4G7A, magenta) [67] and TaCA (PDB code 4C3T, blue) [62]. TaCA dimer was generated using PISA program (http://www.ebi.ac.uk) [71] on the crystallographic coordinates (PDB entry 4C3T).

The SazCA crystallographic structure was recently reported by our group [70], enriching the structural information available on α-CAs from thermophilic organisms. Analysis of this structure revealed the classical dimeric arrangement of bacterial α-CAs [62,67,69] and a substantial similarity with SspCA structure (Figure 4), as expected on the basis of the high sequence identity between the two enzymes (61.3%) [67]. However, a more detailed comparison allowed the identification of minor differences probably responsible of the difference in catalytic activity between SspCA and SazCA. Indeed, although most of the residues of the active site are conserved in the two enzymes, the substitution of the SspCA residues Glu2 and Gln207, located on the rim of the cavity, with His2 and His207 in SazCA, has been proposed to be responsible of the higher SazCA catalytic activity. These mutations might affect the pK_a_ of His64 and consequently its ability to transfer the proton [70]. These results offer interesting prospects for the design of CA variants showing higher stability and catalytic activity than all other α-CAs known to date.

More recent studies by Littlechild’s group reported the biochemical and structural characterization of TaCA, another thermostable and thermoactive α-CA isolated from *Thermovibrio ammonificans* [62]. The enzyme has been overexpressed in *E. coli* and, despite the presence in the sequence of the signal peptide responsible for its *in vivo* periplasmic location, it has been found in the cytosol of the host bacterium. Thus, to mimic the oxidizing conditions of the periplasmic space, a second enzyme form, hereafter indicated as oTaCA, has been expressed and purified in the presence of diamide [62].

Kinetic studies performed on TaCA showed that this enzyme presents a high catalytic activity for the CO_2_ hydration reaction (*k*_cat_ = 1.60 × 10^6^ s^−1^; *k*_cat_/*K*_M_ = 1.6 × 10^8^ M^−1^·s^−1^ at 25 °C and pH 7.5), comparable to that observed for both hCA II and SspCA (Table 1) [62], while only a weak esterase activity has been detected using *p*-nitrophenyl acetate (*p*-NpA) as substrate [45]. Thermal stability studies, performed on both the expressed recombinant forms showed that, after incubation at 70 °C for 1 h, oTaCA retains a higher CO_2_ hydration residual activity with respect to TaCA (residual activity was 90% and 60% for oTaCA and TaCA, respectively) [62]. Finally, a long-term stability analysis showed that TaCA has a half-life of 152 days at 40 °C and of 77 days at 60 °C (Table 2); moreover, after 60 days the enzyme still maintains a high residual activity (91% and 62% at 40 and 60 °C, respectively) [45].

**Figure 5:**
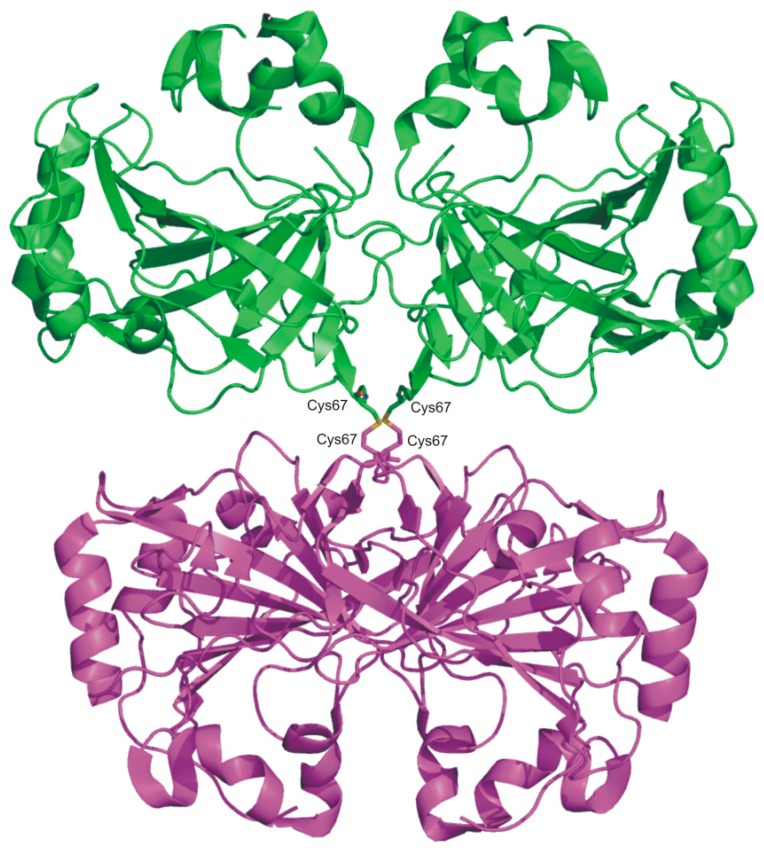
TaCA tetramer structure (PDB code 4C3T) with one dimer shown in green and the other one in magenta. Intermolecular disulfide bonds are also shown.

The analysis of the TaCA crystallographic structure highlighted for this enzyme a fold very similar to that of bacterial homologues previously studied [67,69,70], but a completely new oligomeric arrangement. Indeed, TaCA forms a tetramer (Figure 5), consisting of two dimers, structurally equivalent to those observed for SspCA and SazCA (Figure 4). The two dimers are held together by two intermolecular disulfide bridges and by intersubunit ionic interactions [62]. The increased oligomerization state of TaCA has been suggested to be the feature responsible for the high enzyme thermal stability; this idea is further supported by the observation that other thermostable enzymes present a higher degree of oligomerization with respect to their mesophilic counterparts [72]. It is also worth noting that the two conserved cysteine residues, Cys47 and Cys202, which in SspCA and SazCA form an intramolecular disulfide bond, in TaCA are partially reduced as a consequence of the insufficiently oxidative expression conditions. It has been hypothesized that in oTaCA, this disulfide bond is fully present and responsible of its extra-stability with respect to TaCA [62].

The last thermostable α-CA to be biochemically and kinetically characterized was PmCA, isolated from *Persephonella marina* EX-H1, a bacterium living in the deep-sea hydrothermal vents in the Pacific Ocean and belonging to the order Aquificales [45,46]. The enzyme has been shown to possess both esterase and CO_2_ hydration activity [45,46]; in particular, kinetic parameters for the CO_2_ hydration reaction were determined using stopped-flow spectroscopy at 25 °C and pH 7.8, showing that PmCA is less active with respect to the other thermophilic α-CAs, with *k*_cat_ and *k*_cat_/*K*_M_ values of 3.2 × 10^5^ s^−1^ and 3.0 × 10^7^ M^−1^·s^−1^, respectively (Table 1) [45,46].

Also, in this case thermal stability analysis has been performed, showing that PmCA retains over 80% of its esterase activity after 15 min incubation at temperatures between 60 and 100 °C, while after prolonged incubation times (120 min) at 90 and 100 °C, it retains about 75% and 50% of its activity, respectively [46]. Long-term stability studies, investigated using the CO_2_ hydration assay, showed that after 60 days PmCA has a residual activity of 57% and 27% at 40 and 60 °C, respectively. Moreover, the quantitative evaluation of enzyme half-life at different temperatures has also been reported, showing values intermediate between those observed for SspCA and TaCA (Table 2) [45].

Although no structural information is so far available on PmCA, a comparative amino acid sequence analysis with other members of α-class showed that it presents all highly conserved residues of α-CAs, including the three histidines coordinating the catalytic zinc ion, the proton shuttle His64, and the two cysteines involved into an intramolecular disulphide bridge. Moreover, biochemical studies suggested that, as previously observed for SspCA and SazCA, PmCA exists as a dimer [45].

Altogether these data strongly indicated that these naturally thermostable CAs possess very interesting features suitable for CO_2_ capture technologies. In addition, these enzymes may constitute a basis for obtaining engineered α-CAs with enhanced thermostability and/or catalytic activity.

### 3.2. β-Carbonic Anhydrases

The first β-CA was originally discovered by Neish in 1939 as a constituent of plant leaf chloroplasts [73]; however, the observation that a new CA class non-homologous to α-CAs could exist became evident only subsequently with the determination of the cDNA sequence of *Spinacea oleracea* chloroplast CA [74]. Lately, β-CAs have been found in eubacteria, red and green algae, and in Archaea [75]. All members of the β-CA family need within the active site a catalytic zinc ion, coordinated by three conserved residues (two cysteines and one histidine) and a water molecule/hydroxide ion, to perform catalytic activity; however, although this common structural requirement, these enzymes, in contrast to α-class CAs, exhibit a large structural variability differing for sizes, oligomeric arrangement, domain composition of the monomeric unit, *etc.*, [75].

Among β-CAs, only one thermostable enzyme has been isolated and fully characterized so far, namely Cab from *Methanobacterium thermoautotrophicum*, an archaeon that grows at temperatures ranging from 40 to 70 °C, with an optimal growth temperature of 65 °C [76]. Cab kinetic parameters for the CO_2_ hydration reaction have been determined at 25 °C and pH 8.5, using a stopped-flow spectrophotometer [64]. Data revealed that this enzyme is a less efficient catalyst with respect to the thermophilic α-CAs described in the previous paragraph, with *k*_cat_ and *k*_cat_/*K*_M_ values of 1.7 × 10^4^ s^−1^, and 5.9 × 10^6^ M^−1^·s^−1^, respectively (Table 1). In addition, thermostability studies demonstrated that Cab retains its activity after incubation at temperatures up to 75 °C for 15 min, whereas poor activity is recovered when the enzyme is incubated at temperatures of 90 °C or higher [64], suggesting that Cab is a less suitable candidate to be employed into the harsh conditions of the CCS processes.

**Figure 6:**
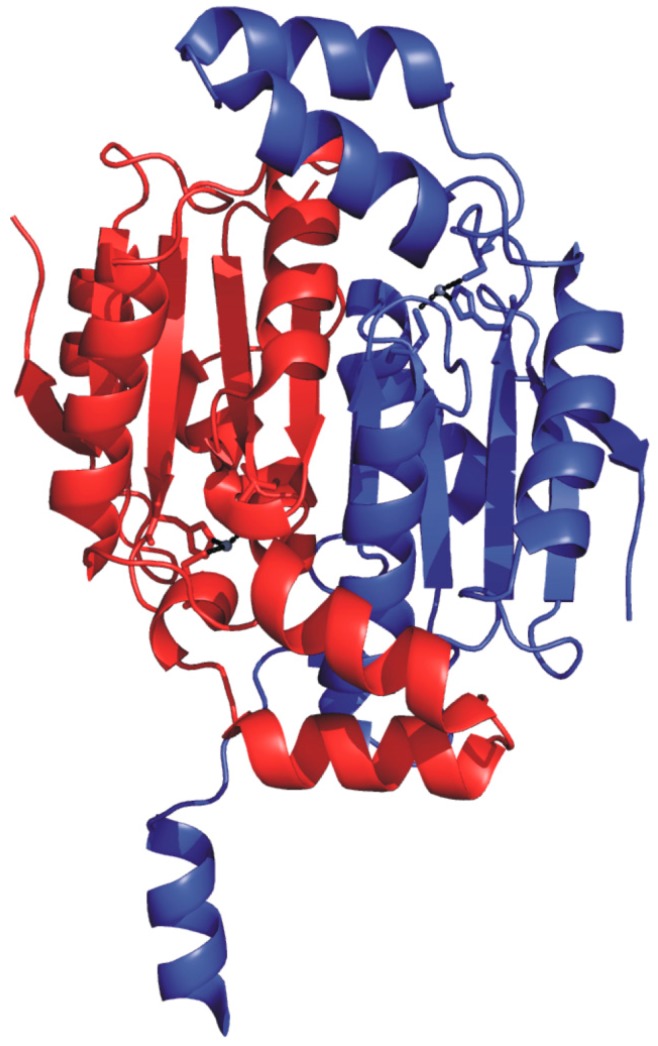
Ribbon representation of Cab dimer structure (PDB code 1G5C) [77] with one monomer colored in red and the other one in blue.

Cab crystal structure has also been reported [77], showing that it exists as a dimer with the typical α/β fold of β-CAs (Figure 6) [78,79,80,81,82,83,84].

**Figure 7:**
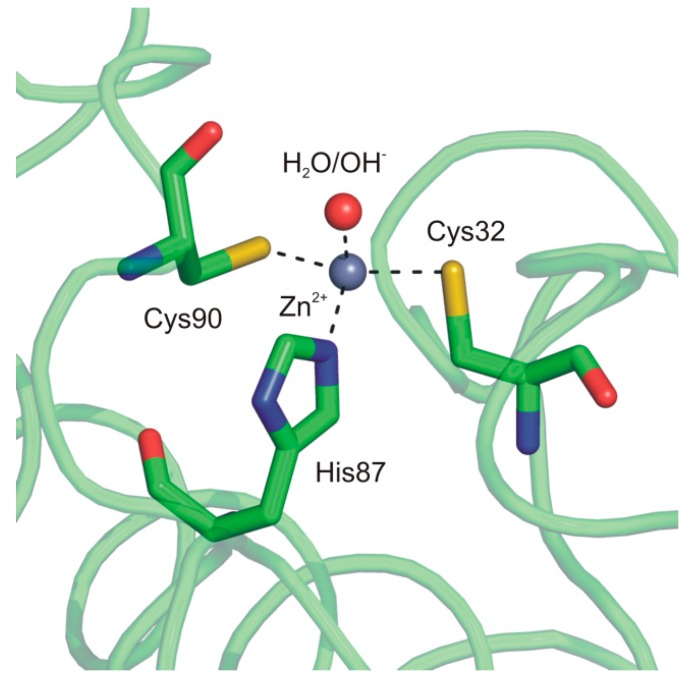
Cab active site representation showing the coordination of catalytic zinc ion.

In particular, the two monomers within the dimer are related by a 2-fold axis and their structure consists of a central β-sheet core composed of five strands. Upon dimer formation, an extended β-sheet core encompassing the entire dimer is formed. Several α-helices pack onto this β structural motif, resulting in a large interface area between the two enzyme subunits (about 2110 Å^2^/subunit) (Figure 6). Interestingly, Cab shows significant structural differences with respect to the other enzymes of β-class in the N-terminus, C-terminus and in the region encompassing residues 90–125. Moreover, it presents a less extended C-terminal region, being the smallest β-CA so far characterized [75,77]. A careful analysis of the Cab active site indicated that, as expected, each monomer contains a zinc ion tetrahedrally coordinated by two cysteines (Cys32 and Cys90), one histidine (His87) and a water molecule/hydroxide ion (Figure 7).

Unfortunately, although a variety of information on Cab enzyme is currently available, no comparative study aimed at identifying structural determinants of its thermostability has been reported so far. Considering that, as mentioned before, Cab does not possess the ideal requisites to be employed in CCS processes, such a study would be highly desirable to provide the basis for the rational design of proteins with improved biotechnological features.

### 3.3. γ-Carbonic Anhydrases

γ-CAs are widely distributed in all three phylogenetic domains of life [85], playing important roles in the global carbon cycle [86]; nevertheless, little biochemical and structural information is currently available on these enzymes [87]. MtCam, a γ-CA isolated from the thermophilic methanoarchaeon *M. thermophila*, is the best characterized member of this enzyme class [88,89].

When overexpressed in *E. coli* and purified aerobically, this enzyme contains zinc in the active site, presents CO_2_ hydration activity but not esterase activity [89] and is moderately thermostable. Indeed, it retains catalytic activity if incubated for 15 min at 55 °C, but only a little activity is recovered when the enzyme is incubated above 75 °C [89]. Kinetic parameters for the CO_2_ hydration reaction, determined by stopped-flow spectroscopy at 25 °C, indicate a catalytic activity comparable to that of Cab but lower with respect to that of thermostable α-CAs previously described (Table 1) [65,90]. Interestingly, in contrast to mammalian α-CAs, this activity doubles when the zinc ion is substituted by cobalt [90]. On the other side, when overproduced in *E. coli* [65] or in *Methanosarcina acetivorans* [66] and subsequently anaerobically purified, the enzyme contains Fe^2+^ in the active site and is 4-fold more active (Table 1) [87]. In these conditions, catalytic activity is rapidly lost after exposure to air, as a consequence of the oxidation of Fe^2+^ to Fe^3+^ and loss of the metal from the active site. Altogether these data provide a convincing evidence that iron is the physiologically relevant metal for this enzyme [65].

**Figure 8:**
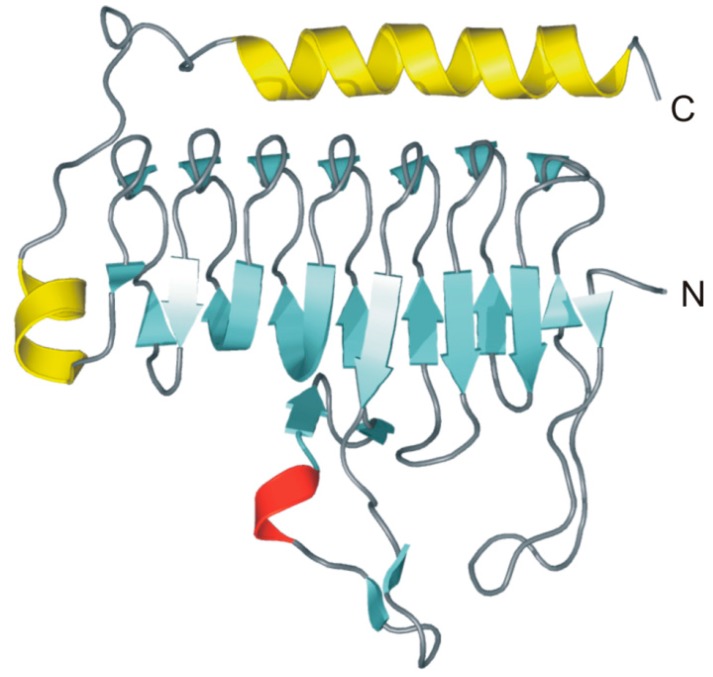
MtCam monomer overall fold (PDB code 1QRG). β-strands are shown in cyan, α-helices in yellow and the 3_10_-helix in red. Secondary structure assignments were obtained from PROMOTIF [93].

The 3D structure of MtCam, in both Zn- and Co-bound forms, has been solved by X-ray crystallography providing interesting structural information on the γ-CA family [91,92]. In particular, in agreement with ultracentrifugation experiments that indicated a trimeric oligomerization state in solution, MtCam crystallizes as a trimer [91], which is formed by three identical monomers of 213 residues [88]. The structure of each monomer within the trimer consists of seven complete turns of a left-handed parallel β-helix with a short α-helix on its top and a second α-helix at the C-terminal portion of the protein, positioned antiparallel to the axis of the β-helix (Figure 8).

**Figure 9:**
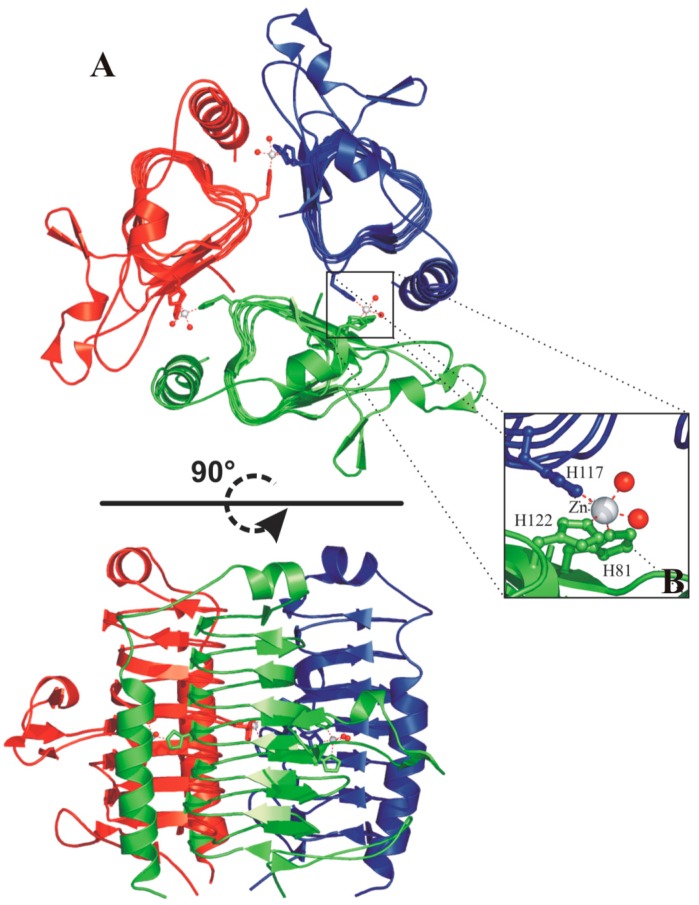
(**A**) Two different views of the MtCam trimer (PDB code 1QRG). The three monomers are reported in green, red and blue. Zinc ions are shown as spheres and their coordinating residues are shown in ball-and-stick representation. Secondary structure assignments were obtained from PROMOTIF [93]; (**B**) enlarged view of the active site of MtCam.

Each face of the β-helix is related to the other two by a 120° rotation, forming an equilateral prism (Figure 9A). Extensive interactions between three β-helices originate the trimer, characterized by a large interface between two adjacent monomers [91]. As a consequence of the trimerization, the three active sites are formed at the interface between two adjacent monomers, with the catalytic metal ion coordinated by three histidine residues, two coming from one monomer (His81 and His122) and the third from the neighboring one (His117) (Figure 9A) [91]. In the case of the zinc-containing enzyme, two water molecules complete the coordination sphere, originating a trigonal bipiramydal geometry (Figure 9B) [92], while in the cobalt containing MtCam the addition of a third water molecule generates a distorted octahedral geometry [92].

Subsequent kinetic and structural analyses of some MtCam mutants [94,95] allowed the identification of residues important for the catalytic mechanism. In particular, Asn73, Gln75 and Asn202 have been demonstrated to be involved in a hydrogen bond network important for the first part of the catalytic process (see Equation (1)), while Glu62 and Glu84 have been proven to be involved in the proton transfer process, during the rate limiting step of the catalytic reaction (see Equation (2)) [94]. Finally, two residues adjacent to the active site, Trp19 and Tyr200, have been identified as contributing to an extended active-site structure distant from the catalytic metal that fine-tunes catalysis [95].

**Figure 10:**
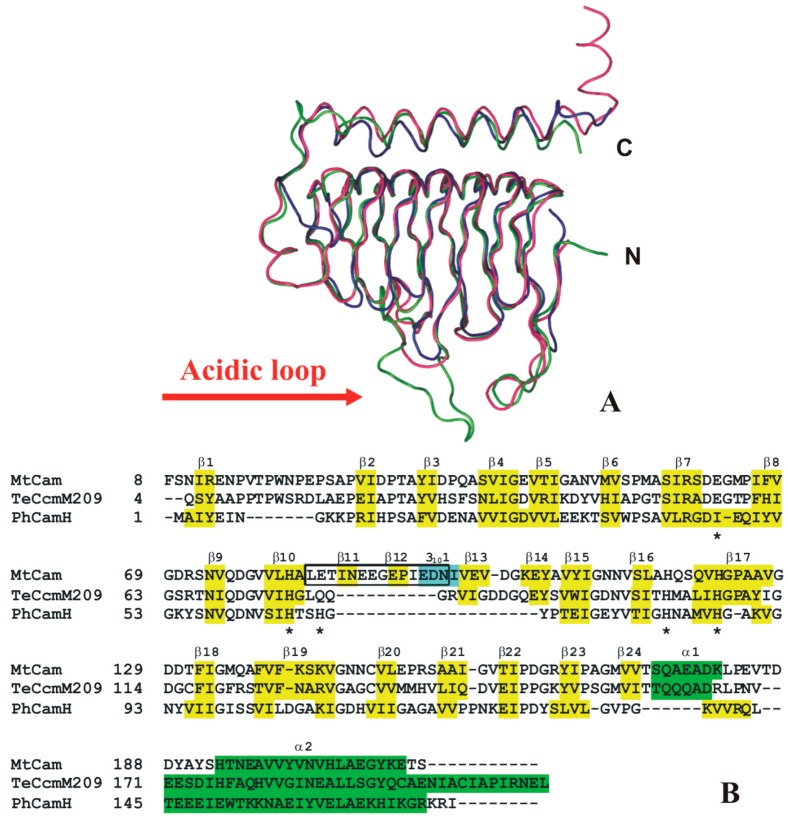
(**A**) Ribbon diagram showing the superposition of MtCam (blue), TeCcmM (green), and PhCamH (red) monomers; (**B**) structure-based sequence alignment of thermostable γ-CAs with known 3D structure. MtCam secondary structure elements are indicated and named according to PROMOTIF analysis [93]. α-helices, 3_10_-helices and β-strands for MtCam (PDB code 1QRG), TeCcmM209 (PDB code 3KWC), and PhCamH (PDB code 1V3W) are highlighted in green, yellow and cyan, respectively. Catalytic histidines, Glu84 and Glu62 of MtCam are indicated with asterisks, while the “acidic loop” (Leu83-Asn96) is boxed.

The crystal structures of two other γ-CAs isolated from thermophilic organisms have been so far reported, namely the γ-CA from the hyperthermophilic archaeon *P. horikoshii* (PhCamH) [96], and the N-terminal domain of the carboxysomal protein CcmM from the thermophilic β-cyanobacterium *Thermosynechococcus elongates* BP-1 (TeCcmM209) [97]. As evidenced by the structural superposition reported in Figure 10A, these two proteins share an overall architecture very similar to that of MtCam. The main differences between these three structures are localized at the N- and C-terminus and in an acidic loop, placed after MtCam strand β10 and containing the proton shuttle residue Glu84 (MtCam numbering). In particular, this loop is much shorter in PhCamH [96] and TeCcmM209 [97] with respect to MtCam (Figure 10A,B) [91].

These structural data, together with the analysis of other γ-CA sequences, have evidenced the existence of two distinct subclasses of γ-CAs. Indeed, in addition to the subclass composed of proteins strictly homologuous to MtCam, a second subclass (named CamH) containing the majority of γ-CAs, can be defined; in this subclass the long acidic loop of MtCam, containing the fundamental proton shuttle residue Glu84 and the catalytically important residue Glu62, is missing (Figure 10). The absence of these residues is quite surprising in view of their role in catalytic reaction and could be indicative of a different function of the members of this subclass [87]. However, despite the absence of these important residues, TeCccM209 presents CA catalytic activity [97]. Unfortunately, no data are available on the catalytic activity of PhCamH [96], creating a major gap in this field to be addressed by future studies. Indeed, since this enzyme is isolated from an archaeon that grows optimally at the temperature of 98 °C, it presents a very high thermostability. The eventual capacity to efficiently catalyze the CO_2_ hydration reaction could make this enzyme a very interesting candidate for biotechnological applications.

## 4. Thermostable Carbonic Anhydrases Obtained by Protein Engineering Techniques

hCA II, the best-characterized human isoform to date, is a very suitable candidate to be used as a biocatalyst for CCS processes. Indeed, this enzyme is easy and cost-effective to be expressed in *E. coli* and purified, it is one of the fastest CAs known (see Table 1), and is very soluble, reaching concentrations of 100 mg/mL. However, its current use in this field is limited by its poor stability in the harsh conditions required by the CCS processes, *i.e.*, temperature from 50 to over 120 °C and high concentrations of organic ions and metals [20,21,22]. For this reason much effort has been dedicated to the development of variants of this enzyme that, although more stable than the native enzyme, would be still able to catalyze the CO_2_ hydration reaction with high efficiency [21,98]. In this context, a recent study from McKenna’s group reported hCA II variants addressing both enzyme thermal stability and catalytic efficiency [21]. In particular, starting from a previous study which identified 10 possible mutations to produce thermostable hCA II variants [99], the substitution of three surface leucine residues (Leu100His, Leu224Ser and Leu240Pro) was used to obtain a triple hCA II mutant (named TS1) with enhanced thermal stability with respect to the wild-type enzyme of about 7 °C. This mutant was then used as starting point to obtain new derivatives with improved catalytic activity. Among these, the variant obtained introducing the contemporary substitutions Tyr7Phe and Asn67Gln retained the same thermal stability as TS1 but showed an improvement in rate constants of proton transfer by about six-fold with respect to the native enzyme [21]. These substitutions were chosen, considering that the double mutant Tyr7Phe/Asn67Gln was previously shown to have a nine-fold increase in the rate of proton transfer compared to the wild-type enzyme [100,101].

The substitution in hCA II of residues 23 and 203 with two cysteines (dsHCA II) [98] to reproduce a disulfide bridge conserved in many members of α-CA class [67,69,70,102,103,104,105,106] was also used to improve hCA II biotechnological properties. Indeed, thermal stability investigations of this variant showed that the melting temperature was enhanced of 14 °C compared to the wild-type enzyme, while the catalytic efficiency was similar to that of native enzyme (*k*_cat_/*K*_M_ of 1.31 × 10^8^ M^−1^·s^−1^) [98].

Finally, considering that protein stability can be improved by increasing the rigidity of surface loops with the inclusion of proline residues [107] and enhancing surface compactness via loop deletion [108,109], novel hCA II variants have been designed [110]. In particular, adopting the first approach, residue Glu234, which is positioned in a surface loop, has been substituted with a proline residue. Thermal stability analysis of this variant indicated an enhanced melting temperature of about 3 °C compared to the wild-type enzyme [110]. On the other side, adopting the second approach, the region encompassing residues 230–240 has been deleted, since it forms an extended surface loop with peculiar destabilizing features [110]. In particular, it is characterized by high thermal fluctuations and the presence of two solvent exposed hydrophobic residues previously shown to affect the thermal stability of the enzyme [21]. The choice was further supported by the observation that in SspCA, one of the most thermostable α-CAs (see Paragraph 3.1), this loop is absent [67]. The thermal characterization of this variant revealed, as expected, an improvement of the melting temperature with respect to the wild-type enzyme.

Altogether these data indicate that the punctual substitutions of critical residues can be effective to obtain active and stable hCA II variants that could be exploited in the harsh conditions required by current industrial processes for atmospheric CO_2_ sequestration.

Since β-CAs are generally characterized by a lower catalytic efficiency compared to the members of the α-class, few studies have so far been performed on their biotechnological applications in CCS processes. However, interesting results have recently been reported by Lalonde and co-workers on some variants of the β-CA isolated from the mesophilic bacterium *Desulfovibrio vulgaris* (DvCA) [41]. This enzyme was chosen since it presented high catalytic activity in 4.2 M *N*-methyldiethanolamine (MDEA), an appropriate solvent for CO_2_ capture processes (see Paragraph 2) [111]. Several variants have been obtained using a directed evolution strategy in combination with the protein activity relationships (ProSAR) algorithm [112]. Among these variants, the most interesting one, containing the substitution of 26 residues with respect to the wild type enzyme, was demonstrated to be highly stable. Indeed, it was able to retain 40% of its activity after being incubated for 14 weeks at 50 °C in 4.2 M MDEA, differently from the native enzyme that had a half-life of 15 min under the same conditions. The variant was also used to assess the rate of absorption of CO_2_ into an aqueous solution of MDEA, showing a net enhancement of the overall rate of CO_2_ capture [41].

## 5. Conclusions

Novel biomimetic strategies are a challenging area of research to develop more ecofriendly CCS processes. In this context, the utilization of CAs has seen increased interest in recent years. Many examples are already available in the literature, especially concerning chemical adsorption and mineral carbonation techniques. However, many limitations to the efficient employment of such enzymes arise from the very harsh conditions typical of the CCS processes. Thus, enzymes active at very high temperatures are of extreme interest. Data reported in this review highlighted that α-CAs isolated from thermophilic organisms, such as TaCA and SazCA, seem to be the most interesting candidates at the moment, both for thermostability features and kinetic properties. Alternatively, thermostable enzymes obtained by applying protein engineering techniques to mesophilic proteins such as hCA II and DvCA also represent an interesting alternative. Altogether, the reviewed data strongly indicate that CAs are of great promise in the field of CO_2_ biosequestration research.
